# Cavitation of Pulmonary Metastases During Enfortumab Vedotin for Metastatic Ureteral Urothelial Carcinoma

**DOI:** 10.1002/iju5.70120

**Published:** 2025-11-16

**Authors:** Fumihiro Ito, Koki Kobayashi, Gaku Hayashi, Shunsuke Kamijo, Takashi Fujita

**Affiliations:** ^1^ Department of Urology Gifu Prefectural Tajimi Hospital Tajimi Japan

**Keywords:** antibody–drug conjugate, enfortumab vedotin, lung cavitation, pulmonary metastasis, upper tract urothelial carcinoma

## Abstract

**Introduction:**

Enfortumab vedotin (EV), a Nectin‐4–targeted antibody–drug conjugate, is active in previously treated urothelial carcinoma. Cavitation of pulmonary metastases is classically linked to squamous histology or anti‐angiogenic/cytotoxic regimens; rarely reported under EV.

**Case Presentation:**

A 69‐year‐old woman with upper‐tract urothelial carcinoma developed pulmonary and hepatic metastases. During EV, multiple lung nodules rapidly cavitated into thin‐walled lesions while liver disease shrank. No infectious symptoms; overall survival was 12 months from EV start.

**Discussion:**

Cavitation likely reflects treatment‐related necrosis once infection and EV‐related pneumonitis are excluded. Diameter‐based criteria can misjudge response when solid nodules cavitate; short‐interval re‐imaging is advisable. Pulmonary response may not ensure systemic control.

**Conclusion:**

Recognizing EV‐associated cavitation as a response pattern may prevent premature discontinuation of effective therapy and unnecessary antibiotics.

**Abbreviations**
ADC: antibody–drug conjugate
CT: computed tomography
EV: enfortumab vedotin
ICI: immune checkpoint inhibitor
MMAE: monomethyl auristatin E
UTUC: upper tract urothelial carcinoma

**Summary**

- Pulmonary metastasis cavitation during enfortumab vedotin should be recognized as a treatment‐effect pattern of tumor necrosis once infection and EV‐related pneumonitis are excluded; it signals response at the involved site but does not guarantee durability across organs.

## 1 Introduction

Urothelial carcinoma of the upper urinary tract (UTUC) is relatively uncommon. Systemic therapy includes immune checkpoint inhibitors (ICI) and antibody–drug conjugates (ADCs). Enfortumab vedotin (EV), a Nectin‐4–targeted ADC, is approved after failure of platinum‐based chemotherapy and immune checkpoint inhibition [1, 2, 3]. Cavitation of pulmonary metastases is an uncommon imaging pattern classically associated with cytotoxic or anti‐angiogenic therapy [4, 5, 6]. To our knowledge, cavitation with EV has not been reported; EV‐related pneumonitis has been reported [7]. We describe a case of metastatic ureteral urothelial carcinoma in which pulmonary metastases developed thin‐walled cavitation during EV therapy.

## 2 Case Presentation

A 69‐year‐old woman with no significant smoking history was referred to our institution with right‐sided flank pain and hematuria. Imaging and ureteroscopic biopsy revealed high‐grade urothelial carcinoma of the right ureter. Clinical staging was cT3N0M0. She received five cycles of neoadjuvant chemotherapy with gemcitabine and carboplatin, followed by laparoscopic radical nephroureterectomy. Postoperative pathology confirmed high‐grade invasive urothelial carcinoma, with negative surgical margins and no nodal involvement (pT3N0).

**FIGURE 1:**
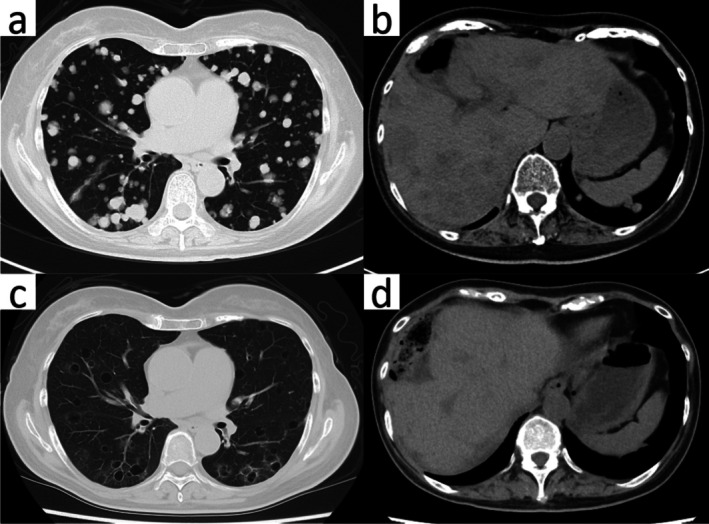
Contrast‐enhanced CT demonstrating disease at baseline and after enfortumab vedotin (EV). (a) Baseline axial lung‐window CT showing solid pulmonary metastases without cavitation. (b) Baseline abdominal CT showing hepatic metastases. (c) After two EV cycles, axial lung‐window CT showing thin‐walled cavitation of pulmonary metastases without air–fluid level. (d) Concurrent shrinkage of hepatic lesions after EV.

**FIGURE 2:**
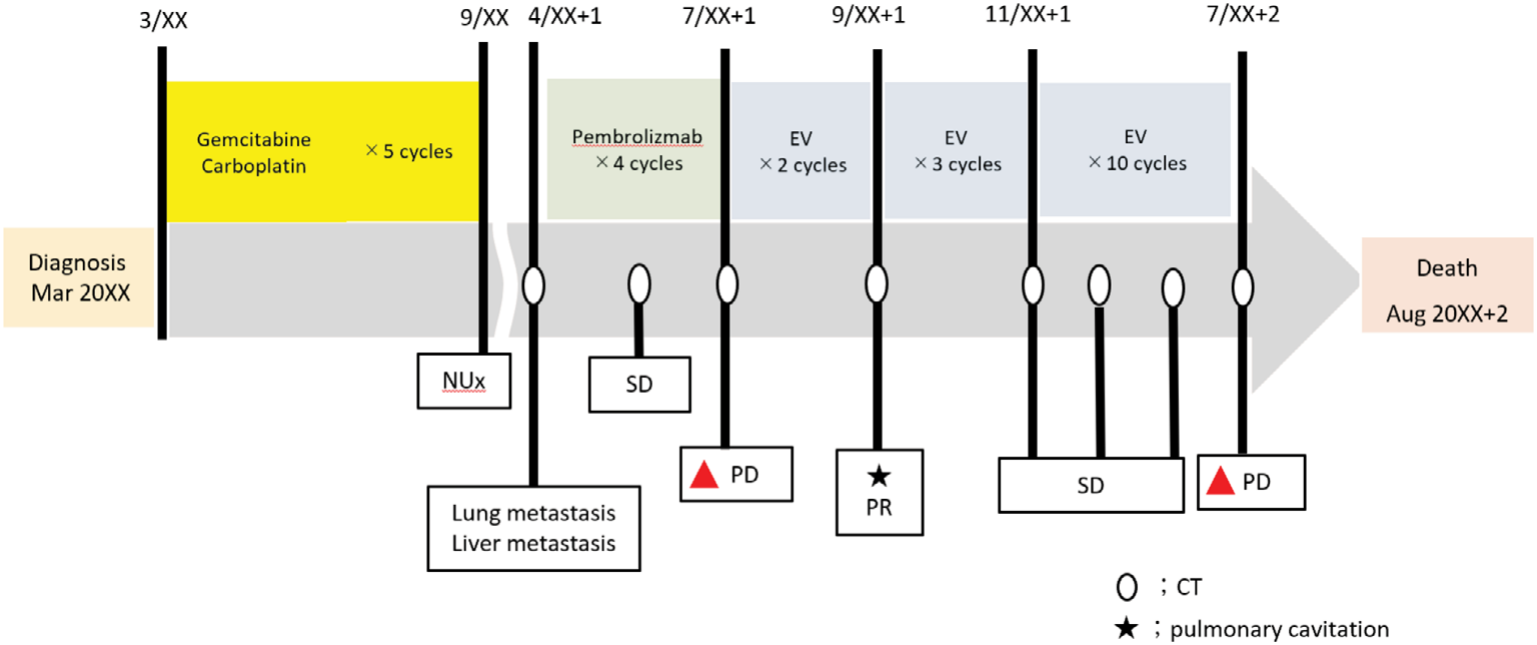
Treatment and assessment timeline. Colored bars denote systemic therapies with cycle counts (Gemcitabine/Carboplatin, Pembrolizumab, Enfortumab vedotin [EV]). Vertical ticks with open circles indicate CT assessments; the wavy gap denotes an interval without imaging. The star marks the first recognition of pulmonary cavitation; red triangles indicate radiologic progression—first after pembrolizumab and later involving lung and liver during EV treatment. The terminal arrow indicates death (date anonymized).

**FIGURE 3:**
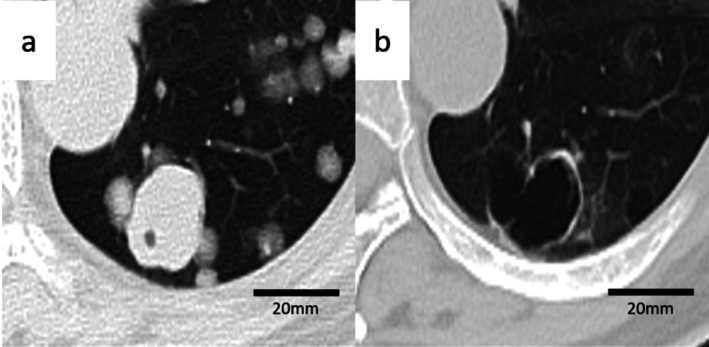
Magnified axial lung‐window CT after two EV cycles showing cavitation of a representative pulmonary metastasis with thin walls and near‐complete loss of solid components; scale bar = 20 mm. (a) Baseline before enfortumab vedotin (EV): Solid round metastatic nodule. (b) After two cycles of EV: The nodule has transformed into a thin‐walled cavity with near‐complete loss of solid components.

**FIGURE 4:**
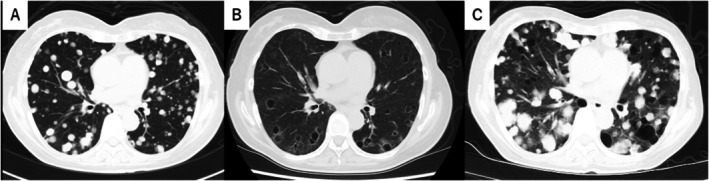
Longitudinal CT of a representative pulmonary lesion: (A) baseline solid metastasis; (B) thin‐walled cavitation after two cycles of enfortumab vedotin (EV); (C) subsequent progression on July XX + 2, showing re‐enlargement of the cavitated pulmonary metastasis, illustrating that cavitation at response does not preclude later outgrowth.

Seven months postoperatively, surveillance contrast‐enhanced computed tomography (CT) identified new pulmonary and hepatic metastases (Figure 1a,b). Pembrolizumab was initiated as second‐line therapy but discontinued after four cycles due to disease progression. Third‐line EV was given at 1.25 mg/kg on days 1, 8, and 15 of a 28‐day cycle, per pivotal dosing [1, 2, 3] (Figure 2). After two cycles, CT showed thin‐walled cavitation of previously solid pulmonary metastases (Figures 1c and 3) with concomitant shrinkage of hepatic lesions (Figure 1d). While pulmonary nodules cavitated, hepatic metastases exhibited size reduction without cavitation on serial contrast‐enhanced CT. Figure 4 shows the lesion at baseline, response, and progression; the patient was afebrile without hemoptysis or inflammatory marker elevation. Cavitation was attributed to EV‐induced tumor necrosis. She continued EV with stable disease for several months. Subsequently, radiologic progression was documented on July XX + 2, characterized by unequivocal enlargement of lung and hepatic metastases. EV was discontinued, and the patient died 12 months after EV initiation.

## 3 Discussion

This case documents a radiologic response pattern—cavitation of pulmonary metastases—observed during enfortumab vedotin (EV) treatment for UTUC. Although cavitation is a well‐recognized phenomenon associated with several cytotoxic and anti‐angiogenic agents [4, 5, 6, 7] its occurrence under a Nectin‐4–targeted ADC remains rarely reported. This broadens known ADC response patterns in urothelial carcinoma.

Although site‐specific EV activity remains debated, UTUC frequently expresses Nectin‐4 [8]. In our patient, EV induced an early radiologic response, while overall survival (~12 months from EV start) fell within the expected range for previously treated metastatic urothelial carcinoma [1, 2, 3]. Cavitation may represent a treatment‐effect pattern consistent with tumor necrosis when infection and EV‐related pneumonitis are clinically and radiologically excluded. In our case, the absence of fever, leukocytosis, inflammatory marker elevation, and hemoptysis argued against infection; however, pathological or microbiological confirmation was not obtained, and therefore this interpretation should be regarded as presumptive. While cavitation can reflect a short‐term therapeutic response, it does not necessarily translate into durable disease control or improved survival. In our patient, despite rapid radiologic improvement after EV initiation, overall survival was limited to 12 months, underscoring that cavitation represents a transient morphological response rather than a surrogate of long‐term benefit. Continued surveillance and comprehensive clinical correlation are warranted. Cavitation reflects local effect, not durable control.

Cavitation in metastatic disease is most frequently described in the lungs, reflecting both true biological proclivity and the higher sensitivity of CT to detect air‐containing changes in aerated parenchyma. Extra‐pulmonary cavitation, such as in the liver, is rare [4, 5, 6, 7]. In our patient, hepatic metastases shrank without demonstrable cavitation on contrast‐enhanced CT (Figure 1d), whereas multiple pulmonary nodules evolved into thin‐walled cavities during EV. This pattern supports that cavitation under EV, when it occurs, is predominantly a pulmonary imaging phenomenon, although systematic organ‐level profiling is still lacking.

A key practical pitfall is confusion with infection or EV‐related pneumonitis, especially in patients with prior or ongoing immune checkpoint inhibitor exposure. Clues arguing against infection include absence of fever, leukocytosis, inflammatory marker elevation, or hemoptysis. On CT, thin‐to‐intermediate cavity walls without air‐fluid levels favor treatment‐related necrosis [7, 9]. In our patient, these features were present, and both pulmonary and hepatic metastases achieved early partial response, supporting true antitumor activity despite later progression. Recognizing this pattern can help avoid unnecessary antibiotics and premature discontinuation of active therapy.

EV binds Nectin‐4, internalizes, and releases monomethyl auristatin E (MMAE) causing tumor necrosis [10, 11]. A bystander effect—diffusion of membrane‐permeable MMAE released via a cleavable linker—may further promote central necrosis in heterogeneous tumors [11]. Cavitation as a radiologic manifestation of extensive necrosis is well recognized with anti‐angiogenic therapy [6], supporting the plausibility of this mechanism under EV. Cavitation likely represents treatment effect when infection and pneumonitis are excluded. The exact pathophysiology in pulmonary metastases remains uncertain and warrants systematic radiologic phenotyping and response assessment that accounts for cavitation [6].

Diameter‐based criteria can underestimate response when a solid nodule becomes cavitary with loss of viable rim; brief qualitative documentation (e.g., “cavitation consistent with treatment effect”) is helpful [6, 12]. Short‐interval confirmatory imaging can reduce misclassification when atypical changes appear [6, 12, 13].

Management should be individualized. In clinically stable patients, isolated cavitation with improving or stable symptoms supports short‐interval re‐imaging rather than automatic regimen change. Since cavitated lesions carry risks—hemoptysis, pneumothorax, superinfection—patients should receive counseling and earlier follow‐up (e.g., 4–6 weeks after first detection) [5, 7, 9, 13]. When the clinical picture is equivocal, bronchoscopy or targeted cultures can clarify infection versus treatment effect; prophylactic antibiotics are not indicated without evidence of infection [4, 5].

Our report has limitations. The inference that EV induced tumor necrosis is based on timing and negative infectious evaluation but lacks histologic confirmation. We did not collect serial quantitative metrics (e.g., Hounsfield units of the viable rim or volumetry), standardized time‐to‐cavitation endpoints, lesional Nectin‐4 expression, or pharmacodynamic biomarkers that might link morphology to target engagement [14]. To our knowledge, a focused search of PubMed, Embase, and Scopus found no prior reports of EV‐associated cavitation of pulmonary metastases; related publications describe cavitary lung lesions with other agents or EV‐associated pneumonitis [4, 5, 6, 9].

Prospective registries should capture cavitation as a predefined variable and correlate it with response and survival. Radiomics and translational studies may clarify mechanisms and identify risk factors [14].

In the setting of EV for metastatic urothelial carcinoma, pulmonary cavitation should be interpreted as a treatment‐effect pattern once infection and pneumonitis are excluded. It indicates response at the time of assessment but does not ensure long‐term disease control, underscoring the need for longitudinal follow‐up and careful clinical correlation.

## 4 Conclusion

Recognition of EV‐associated cavitation as a potential treatment‐effect pattern may help clinicians interpret atypical radiologic changes more accurately. However, this observation is based on a single case, and clinical decisions should rely on comprehensive assessment rather than this finding alone.

## Ethics Statement

According to the policy of our institution, formal IRB review is not required for single‐patient case reports that do not disclose identifiable private information.

## Consent

Written informed consent for publication, including images, was obtained from the patient (or her legal representative) and is available to the Editorial Office upon request.

## Conflicts of Interest

The authors declare no conflicts of interest.

## Data Availability

Data sharing not applicable to this article as no datasets were generated or analyzed during the current study.
